# The complete chloroplast genome of *Hippeastrum vittatum* (Amaryllidaceae)

**DOI:** 10.1080/23802359.2020.1827059

**Published:** 2020-10-09

**Authors:** Peiling Li, Maofei Ren, Qingsong Zhu, Yan Zhang, Hanbing Xu, Zhiyong Wang, Songhu Liu, Qin Cheng, Benguo Liang

**Affiliations:** a College of Horticulture, Xinyang Agricultural and Forestry University, Xinyang, China; b Henan Key Laboratory of Tea Comprehensive Utilization in South Henan, Xinyang Agriculture and Forestry University, Xinyang, China

**Keywords:** *Hippeastrum vittatum*, Amaryllidaceae, complete chloroplast genome, phylogenetic analysis

## Abstract

*Hippeastrum vittatum* (L’Hér.) Herb. is a perennial herb in the Amaryllidaceae, which has been used as a medicinal and ornamental plant. Here, we assembled and characterized the complete chloroplast (cp) genome of *H. vittatum* by high throughput sequencing. As a result, the length of the complete cp genome is 158,082 bp with a canonical quadripartite structure, consists of a large single-copy region (LSC) of 86,165 bp, a small single-copy region (SSC) of 18,283 bp, and two inverted repeat (IR) regions of 26,817 bp, each. A total of 137 genes were identified, including 87 protein-coding genes, 42 tRNA genes, and 8 rRNA genes. The phylogenomic analysis was performed based on the complete cp genomes of 30 species, which revealed the closest relationship between *H. vittatum* and *H. rutilum* in the genus *Hippeastrum.*

The genus *Hippeastrum* Herb. contains abundant alkaloids, and is becoming relevant options for neurological disorders and neurodegenerative diseases (Silva et al. 2008). It comprises more than 70 species, distributed in tropical and subtropical regions of South America (Poggio et al. 2007). There were various hybrids of *Hippeastrum* plants, and most of the modern commercial hybrids were derived from *Hippeastrum vittatum* (L’Hér.) Herb. (Robert et al. 2006; Phuong et al. 2014). Here, we assembled the complete chloroplast genome of *H. vittatum* by high throughput sequencing, and analyzed the characteristic of the cp genome. Our result will provide more information for the phylogenetic reconstruction of the genus *Hippeastrum*.

*Hippeastrum vittatum* was planted in Xinyang Agricultural and Forestry University (E114_13, N32_17), Xinyang, Henan, China. Specimens (no. SZG00057319) were stored at the herbarium of Fairylake Botanical Garden, Shenzhen Chinese Academy of Sciences. Fresh leaves were collected and stored in liquid nitrogen, then stored at −80 °C. Total DNA was isolated using the Plant Genomic DNA Kit (Huayueyang, Beijing, China). DNA integrity and concentration were detected by electrophoresis in 1% (w/v) agarose gel and NanoDrop spectrophotometer 2000. Qualified DNA was used for library construction and sequencing on the DNASEQ T7 platform. A total of 45GB raw data (paired-end 150) was generated, and 117 thousand reads were aligned to the chloroplast genome by the organelle assembler NOVOPlasty Version 3.3 (Dierckxsens et al. 2016). *Hippeastrum rutilum* cp genome sequence (MT133568.1) was chosen as a reference. Finally, genome annotation and visualization were conducted on web server CPGAVAS2 (http://www.herbalgenomics.org/cpgavas2; Shi et al. 2019). The complete chloroplast genome sequence of *H. vittatum* was deposited in GenBank (accession no. MT762362).

The size of *H. vittatum* cp genome was 158,082 bp with 37.9% GC content. It formed a typical canonical quadripartite structure, including a large single-copy (LSC) region of 86,165 bp, a small single-copy (SSC) region of 18,283 bp, and two inverted repeat (IR) regions of 26,817 bp. A total of 137 genes were annotated, which consisted of 87 protein-coding genes, 42 tRNAs, and 8 rRNAs. Analysis of introns and exons found that there were 17 genes were splitting genes with introns and exons, *ycf3* and *clpP* contain two introns and three exons, others have one intron and two exons, which is the same with some *Lycoris* species, such as *L. radiata* (Zhang, Shu, et al. 2019) and *L. longituba* (Zhang, Tong, et al. 2019).

A phylogenetic tree was constructed using 30 species distributed in seven families (Figure 1). Their cp genome sequences were downloaded from the NCBI GenBank database, then aligned by MAFFT (Rozewicki et al. 2019). Maximum-likelihood (ML) phylogeny based on the best-fit model of TVM + F+R4 was conducted using IQ-TREE v. 2.0.3 (Nguyen et al. 2015). The best-fit model was chosen by ModelFinder (Kalyaanamoorthy et al. 2017) according to the Bayesian information criterion (BIC). The robustness of the topology was estimated using 1000 bootstrap replicates. The result showed the closest relationship between *H. vittatum* and *H. rutilum* in the genus *Hippeastrum*, which was grouped on one branch and near to the genus *Lycoris* in the Amaryllidaceae.

**Figure 1.**
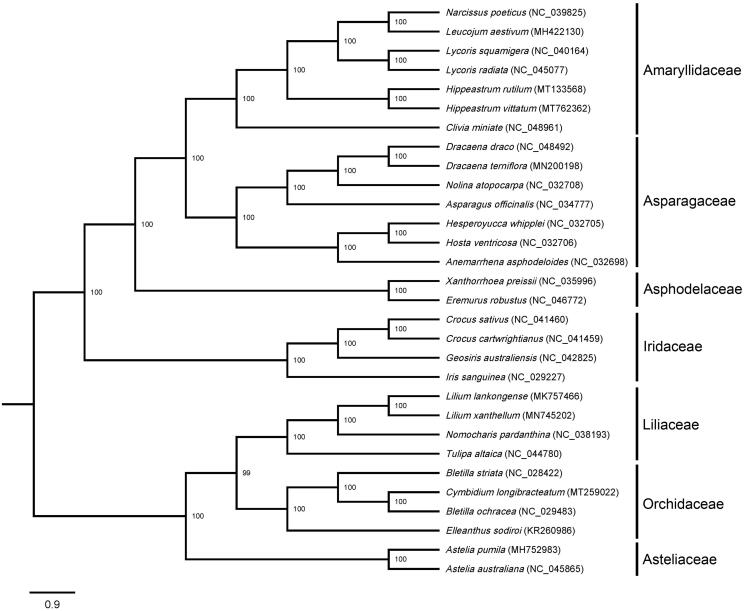
Phylogenetic analysis based on the complete cp genome sequences of 30 species, revealed the closest phylogenetic relationship between *H. vittatum* and *H. rutilum*. The bootstrap values were shown on the nodes, the species and Genbank accession number were shown at the end of each branch.

## Data Availability

The data that support the findings of this study are openly available at https://www.ncbi.nlm.nih.gov/, accession number (MT762362). The raw sequencing data was uploaded in SRA with the reference number PRJNA659134.
